# Steady-State Analysis of Necrotic Core Formation for Solid Avascular Tumors with Time Delays in Regulatory Apoptosis

**DOI:** 10.1155/2014/467158

**Published:** 2014-10-13

**Authors:** Fangwei Zhang, Shihe Xu

**Affiliations:** ^1^College of Urban Railway Transportation, Shanghai University of Engineering Science, Shanghai 201620, China; ^2^School of Mathematics and Statistics, Zhaoqing University, Zhaoqing 526061, China

## Abstract

A mathematical model for the growth of solid avascular tumor with time delays in regulatory apoptosis is studied. The existence of stationary solutions and the mechanism of formation of necrotic cores in the growth of the tumors are studied. The results show that if the natural death rate of the tumor cell exceeds a fixed positive constant, then the dormant tumor is nonnecrotic; otherwise, the dormant tumor is necrotic.

## 1. Introduction

The growth of tumors is a highly complex process. To describe this process, mathematical models are needed. A variety of mathematical models for tumor growth have been developed and studied; for example, compare [1–7] and references therein. Most of those models are based on the reaction diffusion equations and mass conservation law. Analysis of such free boundary problems has drawn great interest, and many interesting results have been established; compare [8–14] and references therein.

In this paper, we study the following problem:

(1) 1r2∂∂r(r2∂σ∂r)=ΓσH(r−ρ),0<r<R(t), t>0,

(2) ∂σ∂r(0,t)=0,  σ(R(t),t)=σ∞,0<r<R(t), t>0,

(3) σ(r,t)=σnec, 0≤r≤ρ(t),

(4) $$\begin{array}{c} \sigma_{r}\left(\rho \left(t\right),t\right)=0,\;\sigma \left(\rho \left(t\right),t\right)=\sigma_{\text{nec}}, \end{array}$$

(5) R2dRdt=∫ρ(t)R(t)s[σ(r,t)−σ~]r2dr −∫ρ(t−τ)R(t−τ)sθ(σ(r,t−τ)−σh)r2dr −∫0ρ(t)sλr2dr, t>0,

(6) $$\begin{array}{c} R\left(t\right)=\varphi \left(t\right),\;-\tau \leq t\leq 0, \end{array}$$

where *r* is the radial variable, *t* is the time variable, the variable *σ*(*r*, *t*) represents the scaled nutrient concentration at radius *r* and time *t*. The variable *R*(*t*) represents the scaled radius of the tumor at time *t* and *ρ*(*t*) represents the scaled radius of the necrotic core of the tumor at time *t*. The three terms on the right hand side of (5) are explained as follows. The first term is the total volume increase in a unit time interval induced by cell proliferation, which is balance between birth and natural death rates of the cells (in the region *ρ*(*t*) < *r* < *R*(*t*)), the birth rate is *sσ*, and the natural death rate is $s\tilde{\sigma }$, where $\tilde{\sigma }$ is a constant. The second term is the total volume decrease (or increase) in a unit time interval caused by regulatory apoptosis, where regulatory apoptosis rate is given by *sθ*(*σ* − *σ*_*h*_); that is, if the local proliferation rate at time *t* − *τ* exceeds (falls below) the critical value *sσ*_*h*_, then there will be an increase (decrease) in local rate of apoptotic cell loss at time *t*, and this increase (decrease) is given by *sθ*(*σ* − *σ*_*h*_), where the magnitude of *θ* indicates the importance of regulatory apoptosis relative to underlying apoptosis: for large values of *θ*, the regulatory mechanism dominates apoptotic cell loss. *s* is a scaling constant. The last term is total volume shrinkage in a unit time interval caused by cell apoptosis or cell death due to aging (in the region 0 < *r* < *ρ*(*t*)); the rate of cell apoptosis is assumed to be constant and does not depend on either *σ*.

The above model is similar to the second model of Byrne [1] but with one modification. The modification is as follows. In Byrne [1], the consumption rate of nutrient is assumed to be a constant Γ, instead of that (1) employed here. In this paper, as can be seen from (1), we assume that the consumption rate of nutrient is proportional to its concentration. This assumption is clearly more reasonable. The reason is as follows. From [1], we know that if the consumption rate of nutrient is assumed to be a constant Γ, then *σ* satisfies

(7) σ(r,R)={σnec,0≤r≤φ(R),σnec−Γφ(R)2+Γφ(R)3r+Γr26,φ(R)<r≤R,

where *φ*(*R*) is the radius of the necrotic core. Therefore, *σ* may be negative for some *φ*(*R*). If one assumes that the consumption rate of nutrient is proportional to its concentration, then *σ* cannot be negative for any *φ*(*R*) (if it has); see (13) and (14) in Section 2.

## 2. Stationary Solutions and the Formation of Necrotic Cores

By rescaling the space variable we may assume that Γ = 1 in (1). For a given *R*, the concentration of nutrient *σ* = *σ*_*R*_(*r*) in the tumor is given by

(8) $$\begin{array}{c} \sigma_{R}\left(r\right)=U\left(r,R\right), \end{array}$$

where *U*(*r*, *R*) is the solution of the problem

(9) 1r2∂∂r(r2∂U(r,R)∂r)=U(r,R)H(U(r,R)−σnec),0<r<R,Ur(0,R)=0,  U(R,R)=σ∞.

Denote *κ* = *σ*_*∞*_/*σ*_nec_. Since *κ* > 1, there exists a unique R* > 0 such that

(10) $$\begin{array}{c} \frac{\sinh R^{*}}{R^{*}}=\kappa . \end{array}$$

**Lemma 1 (see [9]).**

For any *R* > R*, the equation

(11) $$\begin{array}{c} \sinh\left(R-\rho \right)+\rho \cosh\left(R-\rho \right)=\kappa R \end{array}$$

has a unique root *ρ* = *φ*(*R*) in the interval (0, *R*); that is,

(12) $$\begin{array}{c} 0<\varphi \left(R\right)<R,\;R>R^{*}. \end{array}$$

And the solution to the problem (9) is as follows: if  0 < *R* < R*, then

(13) $$\begin{array}{c} U\left(r,R\right)=\frac{\sigma_{\infty }R\sinh r}{r\sinh R}\;\left(0<r\leq R\right),\;\\ U\left(0,R\right)=\frac{\sigma_{\infty }R}{\sinh R}, \end{array}$$

and if *R* > R*, then

(14) U(r,R)={σnec,0≤r≤φ(R),σnecr[sinh⁡⁡(R−φ(R))+φ(R)cosh⁡⁡(R−φ(R))],φ(R)<r≤R.

Denote (*R*_*s*_, *σ*_*s*_(*r*)) as a stationary solution of the problem (1)–(6); then, it satisfies the following equations:

(15) 1r2∂∂r(r2∂σs(r)∂r)=σs(r)H(σs(r)−σnec),σs′(0)=0,  σs(Rs)=σ∞,1Rs2{∫φ(Rs)Rs[σs(r)−σ~]r2dr−∫φ(Rs)Rsθ(σs(r)−σh)r2dr−∫0φ(Rs)λr2dr}=0.

In the rest of this section, we assume that (H)  σh>σ∞>σ~.

The same technique and method can be used to other conditions besides (H), but the results may be different.

**Theorem 2 .**

If $\sigma_{nec}<\tilde{\sigma }+\lambda \leq \sigma_{h}$, then, for any *θ* ∈ (0, *θ*_1_), there exists a unique one positive solution to the equation *f*(*R*) = 0; that is, there exists a unique positive stationary solution to the problem (1)–(6), where $\theta_{1}=\left((\tilde{\sigma }+\lambda )-\sigma_{\text{nec}}\right)/\left(\sigma_{h}-\sigma_{\text{nec}}\right)(>0)$.

**Proof**

By Lemma 1, we find that *R*_*s*_ satisfies the equation

(16) $$\begin{array}{c} f\left(R_{s}\right)=0, \end{array}$$

for 0 < *R* < R*,

(17) $$\begin{array}{c} f\left(R\right)=\left(1-\theta \right)\sigma_{\infty }\frac{R\coth R-1}{R^{2}}-\frac{1}{3}\left[\tilde{\sigma }-\theta \sigma_{h}\right], \end{array}$$

and for *R* > R*,

(18) $$\begin{array}{c} f\left(R\right)=\sigma_{\text{nec}}\left(1-\theta \right)g\left(R\right)-\frac{\lambda }{3}{\left(\frac{\varphi (R)}{R}\right)}^{3}-\frac{1}{3}\left[\tilde{\sigma }-\theta \sigma_{h}\right], \end{array}$$

where

(19) g(R)=1R3[(R−ρ)cosh⁡⁡(R−ρ)+(Rρ−1)sinh⁡⁡(R−ρ)+13ρ3],R>R∗,

and *ρ* = *φ*(*R*). From [9] we know that the function *g* is strictly monotone decreasing for all *R* > R* and *φ*(*R*)/*R* is monotone increasing for all *R* > R*. Using the similar process used in Lemmas 4.1 and 4.2 of [9], one can get the following assertion:* for any θ* ∈ (0,1),* the function f is continuously differentiable and f*′(*R*) < 0* for all R* > 0.

Since, for 0 < *R* < R*, (17) holds, then

(20) lim⁡R→0f(R)=lim⁡R→0(1−θ)σ∞Rcoth⁡R−1R2−13[σ~−θσh]=13[σ∞−σ~−θ(σ∞−σh)],

where we have used the fact lim⁡_*R*→0_(*R*coth⁡*R* − 1)/*R* ^2^ = 1/3. By direct computation, noticing $\sigma_{h}>\sigma_{\infty }>\tilde{\sigma }$, one can get

(21) $$\begin{array}{c} \underset{R\to 0}{\lim}f\left(R\right)=\frac{1}{3}\left[\sigma_{\infty }-\tilde{\sigma }-\theta \left(\sigma_{\infty }-\sigma_{h}\right)\right]>0. \end{array}$$

From [9] we know lim⁡_*R*→*∞*_ *φ*(*R*)/*R* = 1 and lim⁡_*R*→*∞*_(*R* − *φ*(*R*)) = *A*, where *A* is a constant. Then

(22) $$\begin{array}{c} \underset{R\to \infty }{\lim}f\left(R\right)=\frac{1}{3}\left[-\lambda +\left(1-\theta \right)\sigma_{\text{nec}}\right]-\frac{1}{3}\left[\tilde{\sigma }-\theta \sigma_{h}\right]<0 \end{array}$$

for $\sigma_{\text{nec}}<\tilde{\sigma }+\lambda \leq \sigma_{h}$,  *θ* ∈ (0, *θ*_1_)(⊂(0,1)). By the fact that *f*′(*R*) < 0 for all *R* > 0, we have that there exists a unique positive costant *R*_*s*_ that satisfies the equation *f*(*R*_*s*_) = 0. This completes the proof of Theorem 2.

Let *p*(*x*) = (*x*cosh⁡*x* − 1)/*x* ^2^, *x* > 0. From [13], we know that *p*′(*x*) < 0 for all *x* > 0, and

(23) $$\begin{array}{c} \underset{x\to 0+}{\lim}p\left(x\right)=\frac{1}{3},\;\;\underset{x\to +\infty }{\lim}p\left(x\right)=0. \end{array}$$

Since, for $\sigma_{h}>\sigma_{\infty }>\tilde{\sigma }$ and $0<\theta <\tilde{\sigma }/\sigma_{h}$,

(24) $$\begin{array}{c} 0<\frac{\theta \sigma_{h}-\tilde{\sigma }}{\sigma_{\infty }\left(\theta -1\right)}<1, \end{array}$$

then there exists a unique positive solution *x* = *R* ^*c*^ such that

(25) $$\begin{array}{c} p\left(x\right)=\frac{\theta \sigma_{h}-\tilde{\sigma }}{3\sigma_{\infty }\left(\theta -1\right)}. \end{array}$$

**Theorem 3 .**

Assume that condition (H) and $0<\theta <\tilde{\sigma }/\sigma_{h}$ are satisfied. Then the following assertions hold:

- if *R* ^*c*^ ≤ R*, then the dormant tumor ensured by Theorem 2 does not have a necrotic core;
- if *R* ^*c*^ > R*, then the dormant tumor ensured by Theorem 2 has a necrotic core and the radius of the necrotic core is equal to *φ*(*R*_*s*_).

**Proof**

By (17) and (25) one can get that if *R* ^*c*^ ≤ R*, then *f*(*R* ^*c*^) = 0. It follows that (*R*_*s*_, *σ*_*s*_(*r*)) = (*R* ^*c*^, *U*(*r*, *R* ^*c*^)) is the stationary solution of the problem (1)–(6). Since *R* ^*c*^ < R* implies that *U*(*r*, *R* ^*c*^) > *U*(0, *R* ^*c*^) ≥ *σ*_nec_ for 0 < *r* ≤ *R* ^*c*^, we can get that the dormant tumor does not have a necrotic core. If *R* ^*c*^ > R*, then from (17) and (25) and the fact that *p*′(*R*) < 0 we can get *f*(*R*) > 0 for 0 < *R* < R*. Then the solution *R*_*s*_ to the equation *f*(*R*) = 0 satisfies *R*_*s*_ > R*. Consequently, in this case, the stationary solution (*R*_*s*_, *σ*_*s*_(*r*)) = (*R*_*s*_, *U*(*r*, *R*_*s*_)) satisfies *σ*_*s*_(*r*) = *σ*_nec_ for *r* ≤ *φ*(*R*_*s*_). Thus, the dormant tumor has a necrotic core with radius *r* = *φ*(*R*_*s*_). This completes the proof of Theorem 3.

Denote

(26) $$\begin{array}{c} q\left(R\right)=\frac{\sinh R}{R},\;R>0. \end{array}$$

By the fact that *q*(R*) = *κ* = *σ*_*∞*_/*σ*_nec_ and *q*(*R*) is strictly monotone increasing for *R* > 0, we have R* = *q* ^−1^(*κ*). Since

(27) $$\begin{array}{c} p\left(R^{c}\right)=\frac{\theta \sigma_{h}-\tilde{\sigma }}{3\sigma_{\infty }\left(\theta -1\right)}, \end{array}$$

by the fact *p*′(*x*) < 0, one can get that the condition *R* ^*c*^ ≤ R* is equivalent to the following condition:

(28) $$\begin{array}{c} \tilde{\sigma }\geq {\tilde{\sigma }}^{*}=3\left(1-\theta \right)\sigma_{\infty }p\left(q^{-1}\left(\kappa \right)\right)+\theta \sigma_{h}, \end{array}$$

and the condition *R* ^*c*^ > R* is equivalent to the condition $\tilde{\sigma }<{\tilde{\sigma }}^{*}$.

From the above analysis, in view of biology sense, the meaning of Theorem 3 is as follows.

If the natural death rate is large enough such that $\tilde{\sigma }\geq {\tilde{\sigma }}^{*}$, then the dormant tumor is nonnecrotic, and if $\tilde{\sigma }<{\tilde{\sigma }}^{*}$, then dormant tumor is necrotic.

From [9], we know the function *ηp*(*q* ^−1^(*η*)) is strictly monotone increasing for *η* > 1 and *ηp*(*q* ^−1^(*η*)) > 1/3 for *η* > 1. Then we can get the following.

Increasing the nutrient supply *σ*_*∞*_ from surface will increase the threshold value of ${\tilde{\sigma }}^{*}$. This implies that the dormant tumor furnished with a small number of nutrients can possibly be nonnecrotic, whereas the dormant tumor furnished with a large number of nutrients can possibly be necrotic.
